# Nature of Cations Critically Affects Water at the Negatively Charged Silica Interface

**DOI:** 10.1021/jacs.2c02777

**Published:** 2022-10-23

**Authors:** Johannes Hunger, Jan Schaefer, Patrick Ober, Takakazu Seki, Yongkang Wang, Leon Prädel, Yuki Nagata, Mischa Bonn, Douwe Jan Bonthuis, Ellen H. G. Backus

**Affiliations:** † Department for Molecular Spectroscopy, Max Planck Institute for Polymer Research, Ackermannweg 10, 55128Mainz, Germany; ‡ Institute of Theoretical and Computational Physics, Graz University of Technology, Petersgasse16/II, 8010Graz, Austria; § Faculty of Chemistry, Institute of Physical Chemistry, University of Vienna, Währinger Strasse 42, 1090Vienna, Austria

## Abstract

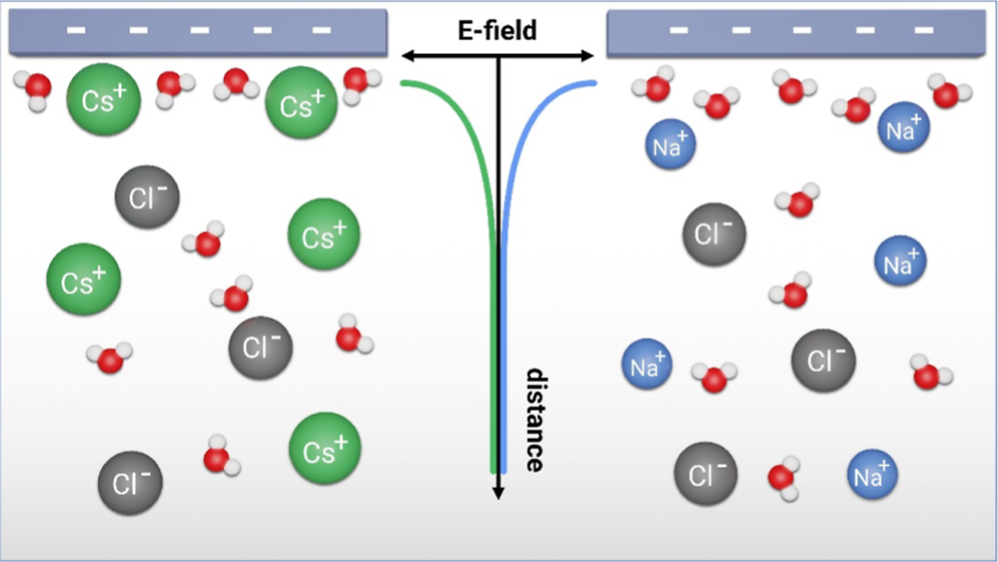

Understanding the collective behavior of ions at charged surfaces is of paramount importance for geological and electrochemical processes. Ions screen the surface charge, and interfacial fields break the centro-symmetry near the surface, which can be probed using second-order nonlinear spectroscopies. The effect of electrolyte concentration on the nonlinear optical response has been semi-quantitatively explained by mean-field models based on the Poisson–Boltzmann equation. Yet, to explain previously reported ion-specific effects on the spectroscopic response, drastic ion-specific changes in the interfacial properties, including surface acidities and dielectric permittivities, or strong ion adsorption/desorption had to be invoked. Here, we use sum-frequency generation (SFG) spectroscopy to probe the symmetry-breaking of water molecules at a charged silica surface in contact with alkaline metal chloride solutions (LiCl, NaCl, KCl, and CsCl) at various concentrations. We find that the water response varies with the cation: the SFG response is markedly enhanced for LiCl compared to CsCl. We show that within mean-field models, neither specific ion–surface interactions nor a reduced dielectric constant of water near the interface can account for the variation of spectral intensities with cation nature. Molecular dynamics simulations confirm that the decay of the electrochemical potential only weakly depends on the salt type. Instead, the effect of different salts on the optical response is indirect, through the reorganization of the interfacial water: the salt-type-dependent alignment of water directly at the interface can explain the observations.

## Introduction

The silica–water interface has been studied extensively as a prototypical mineral interface with surface-specific spectroscopic techniques such as X-ray photoelectron spectroscopy (XPS)^1−3^ and nonlinear spectroscopies such as second harmonic generation (SHG)^4−11^ and sum frequency generation (SFG)^12−23^ spectroscopy. Nonlinear spectroscopies are particularly suited for the study of interfaces, given their intrinsic sensitivity to interfaces, and more specifically, broken symmetry. The charge at the surface serves to align and polarize water molecules near the interface, resulting in symmetry breaking. With SHG—non-resonant frequency doubling of light—it is sometimes challenging to differentiate between different possible origins of the signal: the symmetry is also broken on the silica side of the interface.^24^ In contrast, SFG signals can be enhanced due to resonances with water vibrations, and are therefore exclusively sensitive to the electrolyte. Irrespective of this difference, both methods have provided detailed insights into the microscopic nature of the silica–water interface. For example, pH-dependent studies have revealed that the negative surface charge of silica results from deprotonation of 2–3 types of silanol groups with different acidities.^5−8^ For neutral pH, it has been shown that the second-order nonlinear response of water at the silica surface is highly dependent on the ionic strength of the solution.^9,13,14,16,19,23,25^ Qualitatively, this dependence has been rationalized with the Gouy–Chapman description of the surface potential decay within the electrical double layer (EDL).^9,19,26^ Yet, the measured signals in both, SHG and SFG, experiments comprise the overall signals from different probing depths, which can interfere constructively and destructively,^14,21,26^ making quantitative analysis challenging. Phase-resolved SFG studies have confirmed a marked variation of the phase of the detected signals depending on detection frequency, ν, indicating that the EDL is composed of differently net-oriented water species.^18,22^ Yet not only does the ionic strength of the electrolyte affect the structure of the EDL but also—in line with what has been found for other interfaces^27−29^ —the nature of the ions in the double layer:^5−8,20^ indeed, the nature of the cations in the electrolyte has been reported to markedly affect the intensity of the detected SHG^5,7^ and SFG^20^ intensities and ion-specific trends have been found to strongly depend on the pH of the aqueous solution. To explain such ion-specific effects in SHG experiments, rather dramatic changes in the interfacial permittivity had to be invoked to yield quantitative agreement with mean-field models.^9^ Alternatively, ion-specific acidities of silica’s surface silanol groups,^5^ or extended sizes of ions’ hydrations shells^3^ have been proposed to explain ion-specific interfacial behavior. Here, we present a systematic SFG study on how the different-sized ions alter the EDL composition and the decaying surface potential associated with it. By interrogating the concentration dependence of the SFG response for different alkali halides, we demonstrate that the decay of the potential is rather insensitive to the nature of the electrolyte ions. However, different structures and polarization of water at the very interface due to the charged interface and the ions gives rise to drastically different SFG responses for different ions.

## Results and Discussion

### Nature of the Cation Markedly Affects SFG Intensities

**Figure 1:**
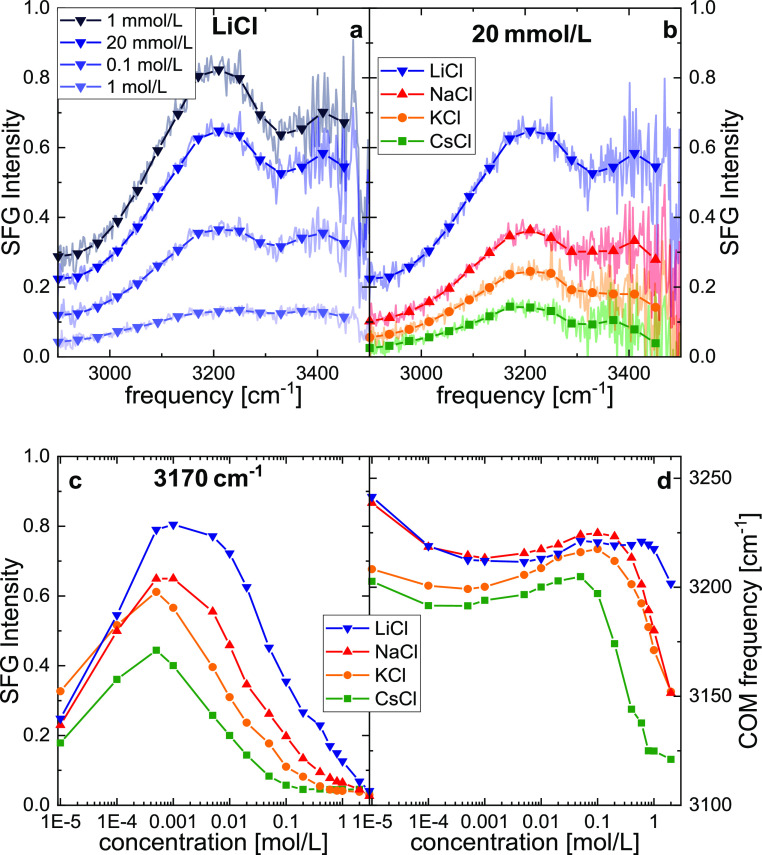
SFG intensity spectra (ssp) at O–H stretching frequencies for the silica water interface with (a) varying concentrations of LiCl and with (b) varying nature of 20 mmol/L salt. Shaded lines show experimental spectra, normalized to the intensity spectra of gold in contact with silica. Symbols show intensities averaged over 20 pixels of the CCD camera (∼40 cm^–1^). (c) SFG response of the H-bonded O–H-stretch vibration at ∼3170 cm^–1^ as a function of salt concentration. (d) Center of mass frequency of the spectra as a function of salt concentration.

In general, the total SFG response of water in front of a charged surface stems from water molecules for which the average centrosymmetry is broken near the interface. For charged interfaces, like, for example, the silica–water interface, the symmetry of water is broken due to the presence of an interfacial electric field^14,19^ and the SFG vibrational response shows a broad, structured band at O–H stretching frequencies (Figure 1a). The interfacial fields can be efficiently screened by the presence of electrolytes. As a result, the measured SFG (or SHG) intensities markedly depend on ionic strength of the electrolyte: the overall SFG intensity of H-bonded water near neutral pH in front of a silica surface decreases with the increase in concentration of LiCl at >1 mmol/L (Figure 1a). Yet, the recorded SFG intensities also markedly depend on the nature of the salt in the aqueous subphase: with the increase in size of the cation from LiCl to CsCl, the magnitude of the SFG response decreases at a concentration of 20 mmol/L salt (Figure 1b)—similar to what has been reported at a higher salt concentration of 500 mmol/L around neutral pH.^20^ For all further analysis, we average the recorded SFG intensities over a frequency range of ∼40 cm^–1^ (symbols in Figure 1a,b). As can be seen from the concentration dependence of these averaged intensities, exemplarily shown at ∼3170 cm^–1^ for all salts in Figure 1c, the SFG intensities as a function of ionic strength exhibit a very similar shape, irrespective of the details of the cation. In line with previous work,^14,19,23^ we observe an increase of the SFG signal with the increase in salt content at low concentration (<0.5 mmol/L) and an inverse behavior at high concentration (>1 mmol/L), giving rise to a maximum SFG signal in the millimolar range.

The shape of the *I*_SFG_ curves as a function of concentration (Figure 1c) can be rationalized by only invoking charge screening and optical interference, in the high- and low-concentration regimes, respectively.^14,19,26^ Quantitatively, the total second-order susceptibility, χ_total_^(2)^, which gives rise to *I*_SFG_, can be split into two contributions: an intrinsic signal due to the anisotropy of water molecules in contact with silica at the very interface, which is often assumed to be independent of the interfacial electric field (χ^(2)^) and a contribution that arises from symmetry breaking due to the presence of the interfacial electric field (*E*_DC_(*z*)) often referred to as the χ^(3)^-contribution.^4,14,19,23,30,31^ These two contributions are sometimes interpreted to report on the so-called Stern and diffuse double layers, respectively.^19,23^ Assuming that χ^(2)^ is independent of the electric field and χ^(3)^ is independent of the spatial coordinate, the SFG response can be expressed as
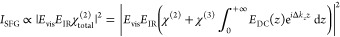
1with *E*_vis_ and *E*_IR_ the electric field intensity of the visible and the infrared laser pulse used in the SFG experiment, respectively. Given that the decay length of the interfacial electric fields can exceed the coherence length of the optical fields (∼1/Δ*k*_*z*_), the phase of the SFG signal depends on the distance *z* from the interface where it is generated. The resulting difference in phase gives rise to interference, which is accounted for by the exponential term in eq 1. Using eq 1 and mean-field approaches such as the Gouy–Chapman theory to determine the ionic strength-dependent *E*_DC_(*z*), the dependence of *I*_SFG_ on bulk ionic concentration (*c*_0_) can be qualitatively modeled: the χ^(3)^-term is negligible at both very low and very high concentrations. At very low concentrations, *E*_DC_(*z*) does not decay within the coherence length, and thus, the second term in eq 1 approaches 0 due to complete destructive interference (see also ref (26)). With the increase in ionic strength, the χ^(3)^-term increases due to reduced destructive interference, because screening reduces the decay length of *E*_DC_(*z*) and the decay length approaches the coherence length of the SFG field. The reduced destructive interference gives rise to the maximum of *I*_SFG_ at millimolar concentrations. Further increasing the salt concentration screens the surface charge over even shorter distances, which results in a decrease of the χ^(3)^-term. At ∼molar concentrations, the contribution of the χ^(3)^-term eventually becomes negligible and only the intrinsic χ^(2)^ is detected, causing *I*_SFG_ to level off at high electrolyte concentrations.

Given that water at different depths has different vibrational responses, the center of masses of the detected SFG spectra, , (Figure 1d), is consistent with the different sensitivities at different ionic strengths: at high salt concentrations the χ^(2)^-term dominates, and water within the sub-nm range is probed. The spectra at high concentrations are therefore sensitive to the structure close to the surface, where ion-specific ion–surface interactions are important. With the increase in size of the cation from LiCl to CsCl, the detected signals at *c*_0_ > 0.1 mol/L undergo an increasing red-shift of the spectral intensity. Therefore, the data shown in Figure 1d point to a cation-specific near-surface structure of water. Conversely, at ∼0.001 mol/L, the χ^(3)^-term dominates, and the spectra interrogate the vibrational response of water at rather long distances from the interface (tens of nm). As such, the response is similar to water’s bulk response, which is hardly affected by low salt concentrations. Consistent with this notion, the center of mass of the spectra is rather similar for all studied salts for low salt concentrations (Figure 1d). We note that in our normalization procedure to quantitatively compare SFG intensities for different salts (see Materials and Methods section), the exact spectral shape is determined by the spectra recorded at 20 mmol/L (Figure 1b). At this concentration, the overall intensity for CsCl is rather low, and, for example, minor non-resonant signals may distort the spectral shape, which may explain the somewhat lower COM values for CsCl. Strikingly, despite the similar spectral shape at 1 mmol/L for the different salts, the magnitude of the detected spectra depends strongly on the nature of the salt: Li^+^ > Na^+^ > K^+^ > Cs^+^. Similarly, the shapes of all measured  curves (Figure 1c) are somewhat similar, yet, the salt concentrations at which *I*_SFG_ plateaus at high concentrations extend over different ranges: while for CsCl, *I*_SFG_ is virtually constant for concentrations above 0.1 mol/L, *I*_SFG_ has not fully leveled off at concentrations as high as 3 mol/L of LiCl.

**Figure 2:**
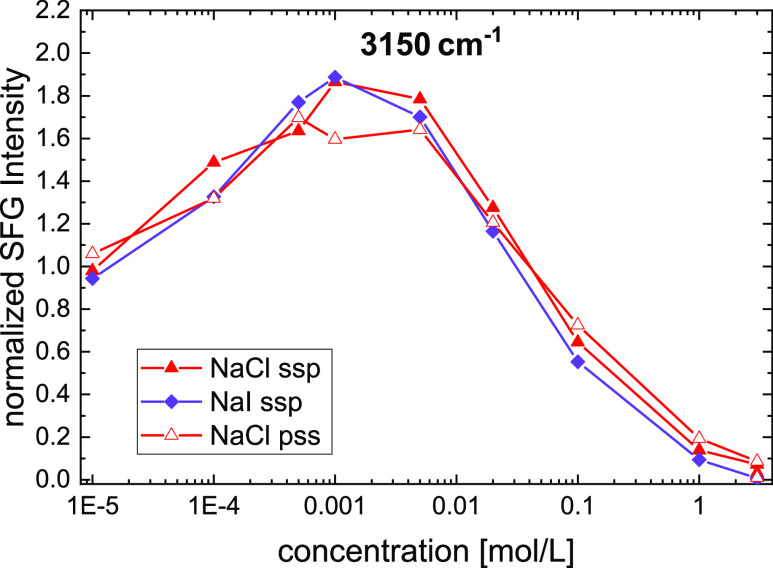
H-bonded O–H-stretch SFG response at ∼3150 cm^–1^ at the silica–water interface with varying salt concentrations. Data for NaCl with ssp polarization combination (solid red symbols), compared to NaCl with pss polarized beams (open red symbols), and to NaI with ssp polarization combinations (purple symbols). Data show averaged values from two independent sets of experiments. SFG intensities were normalized to the intensity in the absence of salt to allow for a better comparison of the ∼four times higher intensity with ssp polarization combination as compared to pss polarizations.

Figure 1 shows that the variation of the cation markedly affects the measured SFG intensities. Although mean-field theories predict a nearly complete depletion of anions near a negatively charged interface,^32^ pH-dependent SHG intensities from silica–water interfaces, normalized to the values at extreme pHs, have suggested that at elevated electrolyte concentrations (*c*_0_ ≥ 100 mmol/L), the anion species has a more dramatic impact on the silica surface charge distribution than the cation species.^6,7^ In contrast to these findings and the marked cation dependence in Figure 1c, we find that the concentration-dependent SFG curve for Na^+^ electrolytes is invariant to the anion, and results for NaCl and NaI virtually overlap at *c*_0_ ≥ 10 mmol/L (Figure 2).

Based on the observation of pH-dependent SFG studies^21^ that the O–H stretch response with pss polarization combination correlates with the surface charge, while spectra with ssp polarization combination (as shown in Figure 1) show a minimum in intensity at ∼pH 7 (much higher than the point of zero charge), it has been suggested that experiments in the ssp configuration are more sensitive to water molecules within the Stern layer, while experiments using pss polarizations probe water in the diffuse layer.^20,21^ Accordingly, we investigate the effect of the polarization combination on the concentration dependence of the SFG intensities studied here. We find for NaCl that employing pss instead of ssp at 3150 cm^–1^ gives rise to a ∼4-times lower response over the whole concentration range (Figure 2, for integrated intensities as used in ref (21) see Figure S1, Supporting Information). Yet, the shape of  is not affected by changing the polarization combination. As such, we conclude that for dilute solutions, ssp and pss provide the same trends upon variation of the ionic strength. As the two polarization combinations are sensitive to water molecules with a different orientation relative to the interface, the fact that the same trend is observed in the ssp and the pss signals shows that the average orientation of water within the probed interfacial region is insensitive to the NaCl concentration.

### Mean-Field Approaches are Insufficient to Explain Intensity Variation

To explore the origin of the ion-specific differences in the SFG intensities, we first consider ion-specific effects on the electrostatic potential in the framework of mean-field models, which allow assessing solvent and ion size effects on the interfacial fields. We focus on a concentration of 10 mmol/L electrolyte, for which Figure 1 suggests that at this concentration the electric field-dependent χ^(3)^-term in eq 1 dominates the overall SFG intensity: 10 mmol/L is well below the concentration at which *I*_SFG_ plateaus for all concentrations, and we neglect the χ^(2)^ term in eq 1 in the following considerations. We note that—as will become apparent below—this neglect leads to an erroneous interpretation of the results. Yet, the concentration is sufficiently high (Debye length ∼ 3 nm) so that the interference term in eq 1 can be neglected. As such, according to eq 1, the SFG intensity is proportional to the square of the integrated electric field, that is, the surface potential, Φ(*z* = 0)
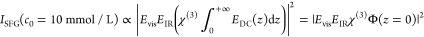
2

Under these assumptions and given that the magnitude of the proportionality constant χ^(3)^ is independent of the nature of the ion, the ∼4 times higher SFG intensity for 10 mmol/L LiCl than for 10 mmol/L CsCl, would imply that the magnitude of Φ(*z* = 0) is ∼twice higher for silica in contact with 10 mmol/L LiCl than with 10 mmol/L CsCl. Similar ion-specific variations in the surface potential have been invoked to explain XPS and SHG experiments.^3^ Within the framework of mean-field approaches, several origins of such an ion-specific increase of the surface potential could be envisioned: (i) increased surface charge density; (ii) finite ion size and other ion-specific effects that limit the approach and/or maximum local concentration of ions; and (iii) reduced interfacial dielectric permittivity of the solvent. In the following, we discuss these three scenarios separately:

(i) Within the Gouy–Chapman theory, the surface potential Φ(*z* = 0) scales with the sinh^–1^ of the surface charge density,^26,33^ which means that Φ(*z* = 0) and the surface charge density are directly proportional for sufficiently low surface charge densities. Thus, to increase Φ(*z* = 0) by a factor of 2, the surface charge density would need to increase by a factor of ∼2 and for higher surface charge densities an even higher relative increase of the surface charge would be required to increase Φ(*z* = 0) by a factor of ∼2. For silica in contact with water, potentiometric titrations have, however, indicated that at neutral pH, the surface charge density is rather insensitive to the presence of alkali chlorides.^34^ More importantly, for negatively charged silica interfaces potentiometric titrations^3,34^ demonstrate reduced surface charge densities in the presence of LiCl, as compared to CsCl. As these trends in surface charge densities contrast with the trend of the SFG intensities, we can dismiss a marked variation in surface charge density as an explanation of the increased SFG intensity for LiCl relative to CsCl.
(ii) The finite ion sizes can limit both the closest approach distance of the ions to the surface and the maximum (local) concentration of ions.^35^ In addition, the local ion density is sensitive to ion-specific ion–surface interactions. To account for both these effects (as opposed to the Gouy–Chapman model, which assumes ions to be point charges with no specific interactions), the underlying Poisson–Boltzmann equation has to be modified.^3^ We use the following formulation^36^
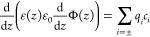
3

where ε(*z*) is the relative permittivity as a function of distance (see below), ε_0_ is the permittivity of free space, and *q*_*i*_ is the charge of the ions. To limit the local ion concentrations *c*_*i*_ to closest packing, *c*_*i*_ is defined as
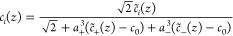
4with *a*_+_ and *a*_–_ as the diameters of the cation and the anion, respectively, and *c*_0_ is the bulk concentration of the electrolyte. The unrestricted ionic concentrations  are given as
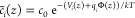
5where *k* is the Boltzmann constant, *T* is the thermodynamic temperature, and *V*_*i*_(*z*) is a distance-dependent potential (see below).

To explore whether the finite ion size can give rise to an increase of the surface potential by a factor of 2 for LiCl as compared to CsCl, we solve eqs 3–5 numerically. Therefore, we assume a constant surface charge density σ = −0.05 C m^–2^ (), typically reported for silica interfaces at neutral pH.^19,34^ ε = 80 is assumed to be independent of distance. To limit the maximum local concentration of ions, we assume *a*_+_ = *a*_–_ = 3 Å as an approximate upper limit of the reported crystallographic radii.^37^ However, we note that for this surface charge density and a concentration of 10 mmol/L (and *V*_*i*_ = 0), the concentration is not limited by closest packing and would only affect such determined surface potentials for *a*_+_ > 8 Å, well beyond the reported sizes of hydrated ions.^37^ Thus, the mean-field modeling suggests that reduced local ion concentrations due to closest packing of ions does not give rise to the ion-specific differences in the surface potentials and thus cannot explain the results shown in Figure 1.

**Figure 3:**
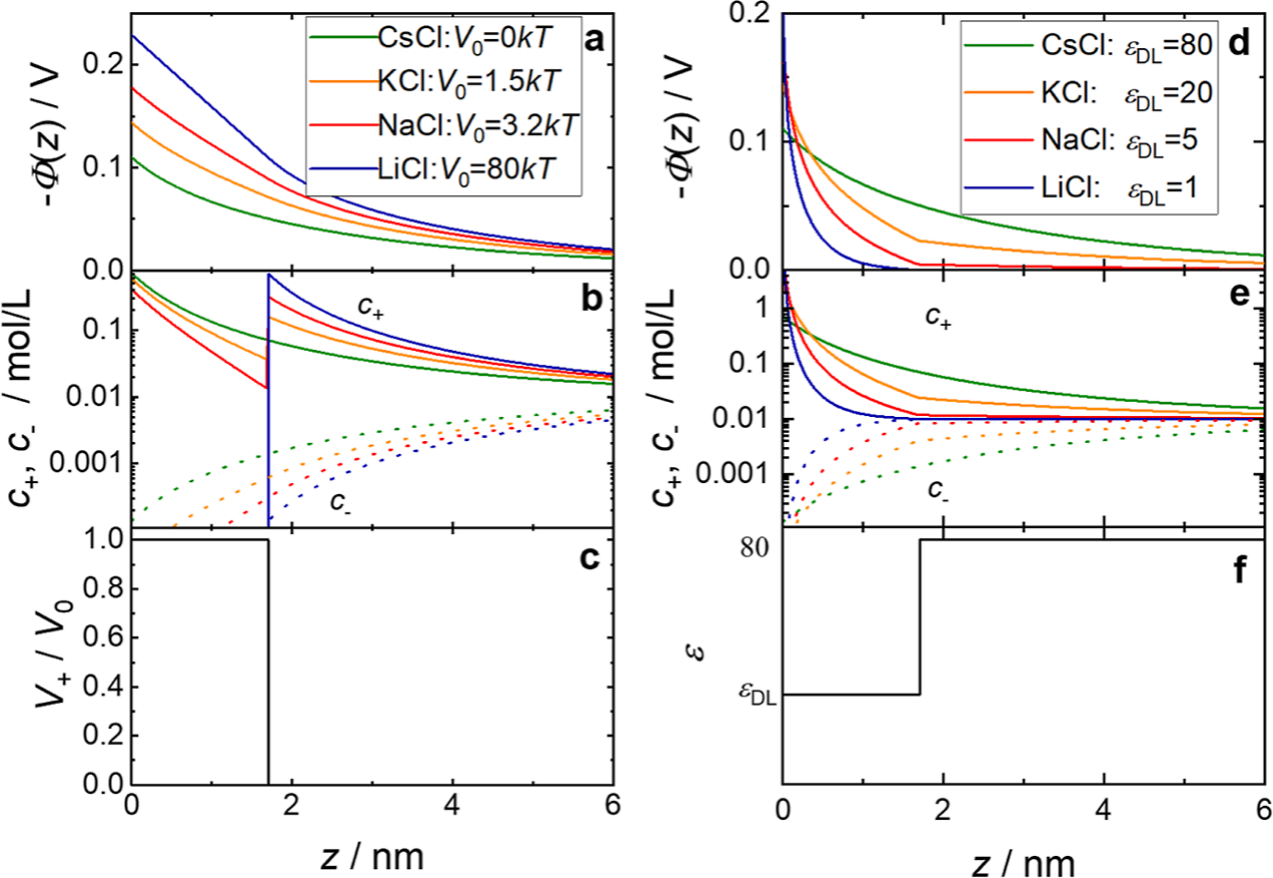
(a,d) Distance-dependent electrochemical potential and (b,e) distance-dependent concentration for cations (solid lines) and anions (dotted lines) obtained by numerically solving eqs 3–5 at 0.01 mol/L of salt using (left) a repulsive potential *V*_+_(*z*) shown in (c) and (right) a distance-dependent dielectric permittivity shown in (f).

In addition to packing effects, the size of the ions may also limit the approach of the ions to the interface and ions may experience a repulsive or attractive ion–surface potential beyond the Coulomb interaction. Together, these effects impose a distance-dependent potential energy term *V*_*i*_(*z*). From molecular dynamics simulations, such potentials of mean force have been suggested to become increasingly repulsive with increasing ionic radii,^38^ and only Li^+^ has been reported to be attracted to the silica interface.^39^ Such potentials extracted from simulations diverge as they approach *z* = 0, yet the effect of the potential on Φ(*z* = 0) is solely determined by the resulting integrated excess ion concentration and does not depend on the functional form of *V*_*i*_(*z*).^40^ Thus, for convenience, we use a square potential with *V*_+_(*z* ≤ 1.7 nm) = *V*_0_ and *V*_+_(*z* > 1.7 nm) = 0. The reason for choosing a width of the potential of 1.7 nm will become apparent below. Because anions are repelled from the negatively charged surface, *V*_–_ hardly affects the solution of eq 3, and we assume *V*_–_ = 0. To explain the relative differences in the SFG intensities, only ion-specific relative potential barriers are relevant, and thus, we arbitrarily set *V*_0_ = 0 for CsCl (Gouy–Chapman limit), which results in a surface potential of Φ(*z* = 0) = −0.11 V (Figure 3a). To achieve a relative increase in Φ(*z* = 0) at *c*_0_ = 10 mmol/L according to the SFG intensities in Figure 1c, the potential barrier has to increase from *V*_0_ = 0 to *V*_0_ ≈ 1.5*kT* for KCl and *V*_0_ ≈ 3.2*kT* for NaCl. Such repulsion of these ions from the silica interface can be rationalized by increasing the ion hydration.^3^ However, in order to increase Φ(*z* = 0) by a factor of 2 (Figure 3a) for LiCl, *V*_0_ has to be set such that Li^+^ ions are fully repelled from the interface over a distance of 1.7 nm [i.e., a length of the box potential of 1.7 nm is required to double Φ(*z* = 0)] (Figure 1b,c). We note that less drastic changes have to be invoked to double Φ(*z* = 0) at higher ionic strengths, for example, repulsion over 1 nm at 50 mmol/L (the concentration used in ref (3)). In ref (3), an increased surface potential due to such strong repulsion has been invoked to result from the strong hydration of Li^+^. This reported increase in potential is in line with some experimentally observed trends in ζ-potentials,^3,41,42^ albeit of different magnitudes,^3,41,42^ and also opposite trends have been reported.^43^ Different silica samples can partially explain different reported values of the ζ potential, but also ion-adsorption has been argued to critically affect the observed interfacial electrostatic properties.^41^ In fact, adsorption of Li^+^ to silica finds support from molecular dynamics simulations^39^ (see also below), from some reported ζ potentials^43^ and from the potential at the outer Helmholtz plane being lowest for Li^+^ at neutral pH.^44^ Irrespective of these partially contrasting literature results, the required strong repulsion for Li^+^ relative to Cs^+^ (which we have arbitrarily assumed to approach the surface barrier-free) that is required to explain the present results appears to be not realistic. As such, surface repulsion also seems implausible as the sole cause for the ion-specific differences shown in Figure 1.

(iii) Lastly, strong hydration of ions and/or interaction of water with the surface can alter the properties of the solvent water, which is often related to a reduction of the dielectric permittivity (dielectric saturation).^9,45,46^ To test whether a decrease in the dielectric permittivity near the interface can give rise to the observed changes in the SFG intensities as shown in Figure 1, we solve eqs 3–5 using a distance-dependent permittivity. We note that the functional form of how ε(*z*) precisely decreases toward the interface negligibly affects the above-discussed increase of Φ(*z* = 0). Rather the value of ε(*z* = 0) determines Φ(*z* = 0) for a constant surface charge density of σ = −0.05 C m^–2^.^19,34^ Thus, we assume the same box function as for the potentials above: ε(*z* > 1.7 nm) = 80 and ε(*z* ≤ 1.7 nm) = ε_DL_ (and *a*_+_ = *a*_–_ = 3 Å; *V*_+_ = *V*_–_ = 0). As can be seen in Figure 3, even for a drop to ε(*z* = 0) = 1, the surface potential increases by only 80%. In order to increase Φ(*z* = 0) by 30 and 60% (as suggested by the SFG data at 10 mmol/L for KCl and NaCl relative to CsCl, respectively; see Figure 1), the permittivity has to decrease to a value of ε(*z* = 0) = 20 and ε(*z* = 0) = 5, respectively (Figure 3d–f). Although such changes have been reported for the effective permeability of strongly confined water,^46^ the large variation in these changes with the nature of the cation appears too drastic to be physically meaningful. In fact, force microscopy experiments^45^ indicate that, while appreciably lower than in bulk, ε is very similar for different electrolytes at *z* = 0. The negligible effect of ions on the dielectric properties of the interfacial water layer has also been confirmed using a combination of molecular dynamics simulations and electrokinetic experiments.^47^

**Figure 4:**
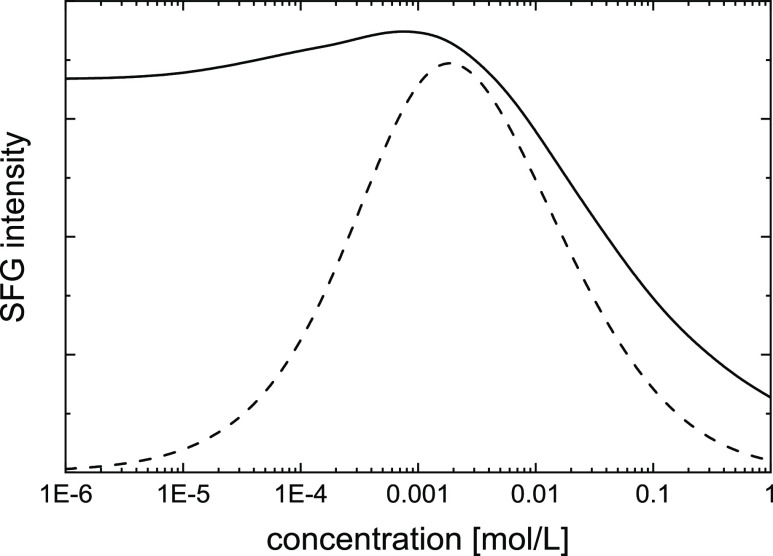
Modeled (eq 2) SFG intensity  using a 1.7 nm repulsive square potential (*V*_0_ = 3.2*kT*) as a function of ionic strength, expressed here, for monovalent ions, as bulk concentration *c*_0_ (solid line). For comparison, we also include the SFG intensity calculated using a mono-exponentially decaying electric field *E*_DC_(*z*) (Gouy–Chapman, dashed line). To illustrate the differently decreasing behavior with the decrease in concentrations of both curves, we take Δ*k*_*z*_ = 1/10 nm.

Based on these considerations, we conclude that the above-described scenarios (i–iii) require rather unphysical changes of the EDL near the interface to explain the observed ion-specific SFG intensities. In line with this notion, atomic force experiments have suggested that the surface potential of silica is only weakly sensitive to the cation size at neutral pH values.^44^ While with a combination of the effects (e.g., repulsion of ions and a decrease of the dielectric permittivity), less dramatic alteration of the interfacial properties would be required to increase the surface potential by a factor of 2, the altered interfacial properties are typically reported to be spatially limited to a few nanometers from the interface. Such spatial limitation to a few nanometers has, however, important consequences for the shape of : the near-surface modulation of the interfacial properties enhances (or reduces) the electric field within these few nanometers (cf. the potential in Figure 3a,d at *z* < 2 nm). As such, at low concentrations, full destructive interference (see eq 1) cannot be obtained, and the SFG intensities as a function of ionic strength become inherently asymmetric (as opposed to the rather symmetric curves in Figure 1c): in Figure 4, we show exemplarily the values of  as obtained from the mean-field modeling for NaCl using *V*_0_ ≈ 3.2*kT* (see above) and Δ*k*_*z*_ = 1/10 nm (Note that the coherence length is much shorter than the experimental one, and just chosen to avoid the time-consuming numerical solution of eqs 3–5 at low ionic strengths). The asymmetric shape of the solid curve in Figure 4 contrasts with the rather symmetric experimental observations. Therefore, the relatively symmetric  curves in Figure 1c suggest that the origin of the strong ion specificity of  cannot be found in ion-specific interfacial effects that drastically change the local electric field. Rather, the symmetric  curves in Figure 1c suggest that the origin of the strongly varying magnitude of *I*_SFG_ for different ions must stem from ion-specific modulation of *I*_SFG_ that also affects the SFG signals generated at distances exceeding the coherence length (>40 nm, see below).

### Molecular Dynamics Simulations Predict Weakly Ion-Specific Interfacial Potentials

To obtain more detailed insights into the ion-specific structure of the double layer, we performed molecular dynamics simulations of silica water interfaces in contact with aqueous solutions of CsCl, KCl, NaCl, and LiCl. Briefly, we model the silica interface using the force field developed by Emami et al.,^48^ with 5% of all silanol groups being deprotonated (∼−41 mC m^–2^ surface charge density). Water was modeled using the SPC/E force field.^49^ For the ions, we have compared various reported force fields,^50−55^ and the details of this comparison are shown in Figure S2, Supporting Information. We simulate for each salt three different background salt concentrations of 1, 0.1, and 0 mol/L in addition to the cations (∼0.1 mol/L) that compensate for the surface charge. From the distance-dependent total charge distribution, ρ_total_(*z*), which can be decomposed into the contributions from the ions, water, and the surface [ρ_total_(*z*) = ρ_ions_(*z*) + ρ_water_(*z*) + ρ_surface_(*z*)], we obtain the electric fields, *E*_*i*_(*z*), as
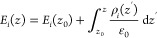
6where the index *i* = total, ions, water, or surface.  is an integration constant and *z*_0_ the center of the silica.

**Figure 5:**
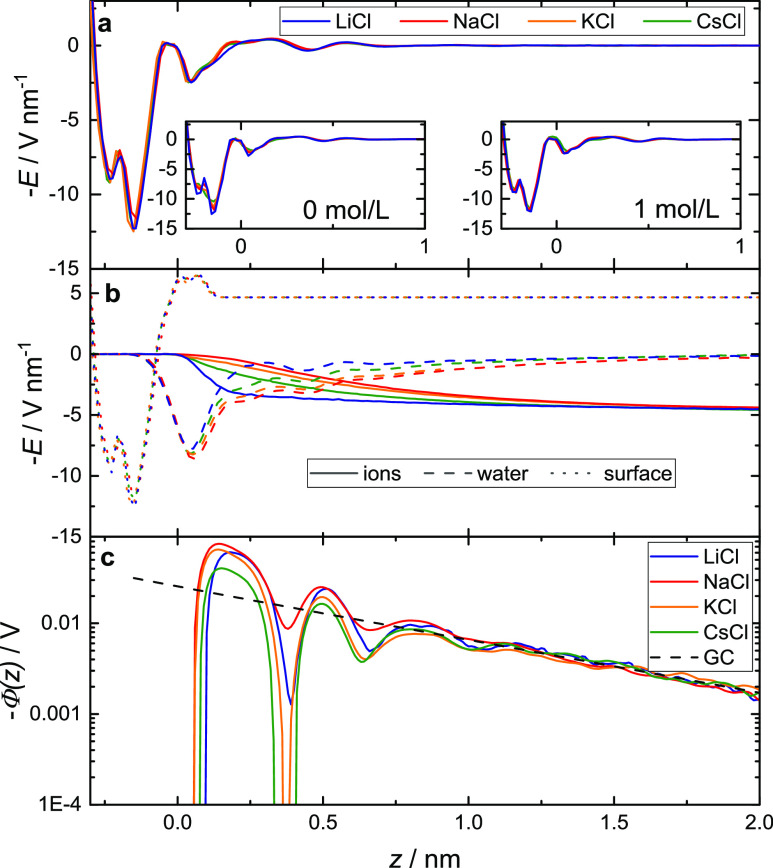
Interfacial electrostatic properties calculated from the simulations. (a) Total electric field at a concentration of 0.1 mol/L (note that curves overlap). Total electric fields with counterions only and at a concentration of 1 mol/L are shown in the inset. (b) Individual contributions from water, ions, and the (silica) surface at 0.1 mol/L. (c) The electrochemical potential at 0.1 mol/L together with the Gouy–Chapman approximation using an effective surface charge density of −28 mC m^–2^ and the dielectric permittivity of SPC/E water ε = 70.^57^ The Gibbs dividing surface of the water is located at *z* = 0.

In Figure 5a, we show the total electric fields, which we find to be rather insensitive to the nature of the ions at all three concentrations. This insensitivity to the ion’s nature suggests that the ion-specific variation of the electric fields, which affects the measured SFG intensities according to eq 1, cannot explain the markedly different SFG intensities shown in Figure 1b,c.

To gain deeper insights into the ion-specificity of the distance-dependent distribution of the ions, we show the individual contributions to the total electric field in Figure 5b. The electric field due to the surface, *E*_surface_(*z*), is exclusively determined by the structure of silica and, as such, independent of the electrolyte. Conversely, the ionic displacement fields, *E*_ions_(*z*) varies with the size of the ion: the ionic displacement fields in Figure 5b increasingly extend into the solution with the decrease in cation size from CsCl to NaCl. Although this longer decay length for Na^+^ as compared to Cs^+^ is at variance with earlier simulation studies of highly charged interfaces^56^ (presumably related to the different charge densities and different force-fields, see the Supporting Information), it is consistent with the larger hydrated radius of Na^+^ as compared to (weakly hydrated) K^+^ and Cs^+^.^3^ For LiCl, the simulations predict the displacement field to decay more rapidly than for the other alkaline metal salts: the data in Figure 5b show a steep decrease of the ionic displacement fields within a distance of ∼0.3 nm from the interface for Li^+^, which is due to ion adsorption at the interface. The adsorption of Li^+^ is consistent with earlier simulation results.^39^ Yet, the significance of this observation should be interpreted with caution, as this adsorptive behavior depends on the choice of the force-field (see Figure S2, Supporting Information) and the force field of Li^+^ fails in reproducing experimental bulk activity coefficients (Figure S3, Supporting Information). Irrespective of these ion-specific differences and the issues related to the Li^+^ force-field, the ion-specific differences in *E*_ions_(*z*) are nearly fully compensated for by the field from water *E*_water_(*z*) (Figures 5b and S2, Supporting Information), resulting in *E*_total_(*z*), being insensitive to the cation.

Therefore, our simulation results show that despite ion-specific differences in the distance-dependent composition of the double layer for moderately high electrolyte concentrations, the total surface field is—for a given concentration—very similar for all salts. This similarity supports the conclusions from the discussion of the mean-field models above: the variation of the surface potentials and surface fields with cation size is too weak to explain the experimentally observed large variations in the SFG intensities. Yet, the simulations show that despite the insensitivity of the net electric field to the nature of the ions, the electric fields due to the ions and water are very different for different ions. The ion-specific differences in *E*_water_(*z*) imply marked differences in the ion-specific near-surface water structure. As a result, the electrostatic potentials, as determined from the MD simulations, strongly deviate from mean-field predictions using, for example, the Gouy–Chapman model at short separations and approach the mean-field limit only at *z* > 1 nm (Figure 5c). Conversely, the MD simulations suggest that the ion-specificity in the response of water at the silica interface is mostly limited to *z* < 1 nm (Figure 5b,c).

### Interference of Bulk and Surface Signals can Explain Cation-Specificity

The above considerations collectively suggest that ion-specificity in the detected SFG intensities cannot be explained based on only interfacial electric fields (i.e., the χ^(3)^-term in eq 1). Moreover, the increase in SFG intensities when increasing the cation size from CsCl to LiCl is caused by effects limited to the very interface (*z* < 1 nm), which is commonly ascribed to the χ^(2)^-term in eq 1. One apparent simplification in the electrostatic considerations in eq 2 is neglecting the response of water molecules at the very surface (χ^(2)^-term in eq 1). Both χ^(2)^ and χ^(3)^ are complex-valued quantities. Hence, the two terms in eq 1 can have a phase difference and can interfere destructively or constructively. An ion-specific interference could give rise to ion-specific variations in the magnitude of the SFG intensity, irrespective of the ionic strength.

**Figure 6:**
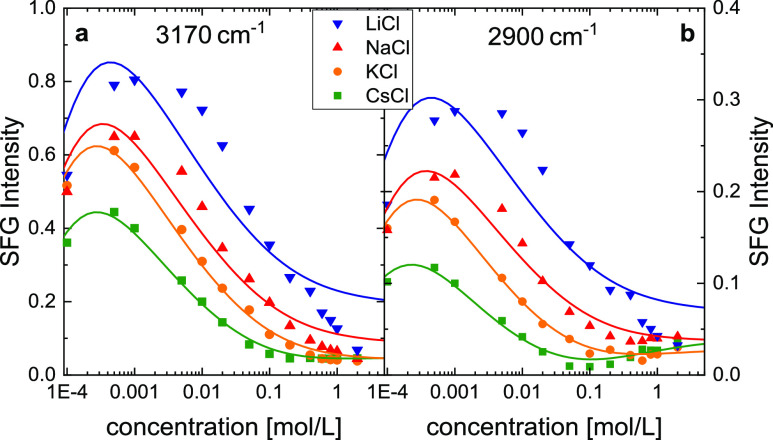
SFG (ssp) response of the H-bonded O–H-stretch vibration at (a) ∼3170 and (b) ∼2900 cm^–1^ for the silica–water interface with varying salt concentrations using different alkali chlorides: LiCl (blue), NaCl (red), KCl (orange), and CsCl (green). Symbols show the experimental data integrated over a ∼40 cm^–1^ range. Solid lines show fits according to eq 7.

To investigate potential interference between the χ^(2)^- and χ^(3)^-terms, we consider the IR frequency-dependent  curves: the experimentally determined  profiles at 3170 cm^–1^ in Figure 6a exhibit a continuous decay of *I*_SFG_ with the increase in ionic strength for all studied salts at *c*_0_ > 1 mmol/L. As such, interference effects due to χ^(2)^ and χ^(3)^ are not directly apparent from the data at 3170 cm^–1^. At 2900 cm^–1^ (Figure 6b), the data are consistent with destructively interfering χ^(2)^ and χ^(3)^ signals: most apparent for CsCl, and somewhat less pronounced for KCl and NaCl, the measured  values decay from a maximum at millimolar concentrations to a minimum at ∼0.1 mol/L after which they increase again toward 1 mol/L. This observed minimum intensity at ∼0.1 mol/L is indicative of destructive interference between the χ^(2)^- and χ^(3)^-terms. To evaluate the interference of both contributions to the SFG intensities, we model the data at all frequencies. For convenience, we assume an exponentially decaying surface potential, which decays according to the Debye Hückel parameter, , so that eq 1 simplifies to^19^
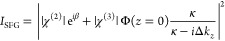
7where we use the absolute values of the interfacial and bulk responses |χ^(2)^| and |χ^(3)^|, respectively, and their relative phase β as adjustable parameters. The wave vector mismatch we estimate from the experimental geometry (angle of incidences for IR: 33°, vis: 37°, and SFG: 36°; refractive index of the electrolyte at vis and SFG frequencies *n*_vis,SFG_ = 1.33 and at IR frequencies *n*_IR_ = 1.409)^19,58,59^ to Δ*k*_*z*_ = 1/42 nm. The ionic strength-dependent surface potential we obtain by assuming a constant surface charge density of σ = −0.05 C m^–2^^19,34^ via .^33^

As shown exemplarily for two infrared frequencies in Figure 6, eq 7 describes the experimental  curves very well for CsCl and KCl. For NaCl, we find minor deviations of the fitted curve from the data points at high ionic strengths. For LiCl, the description of the data with the model is clearly worse, which may be explained by the composition and structure of the interfacial region being altered upon the addition of LiCl, which would result in a concentration-dependent |χ^(2)^| response.

**Figure 7:**
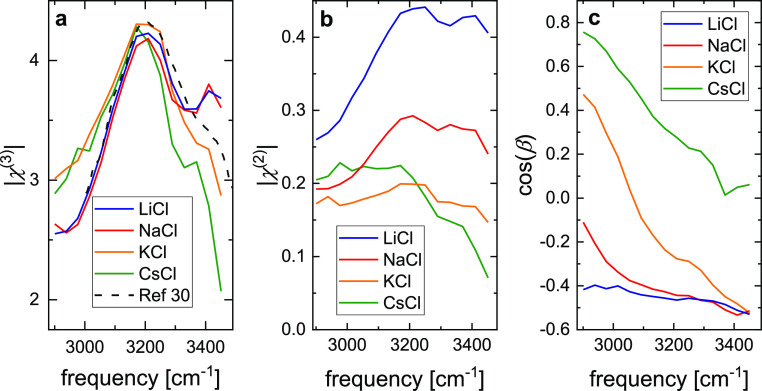
Results for (a) , (b) , and (c)  as a function of IR frequency as obtained from modeling the  data integrated over a ∼40 cm^–1^ range (solid lines in Figure 6) using eq 7 for LiCl (blue), NaCl (red), KCl (orange), and CsCl (green). In panel (a), we also show scaled  spectra for water in contact with a charged lignoceric acid monolayer at pH 6 in the presence of NaCl, as extracted from Figure 2b of ref (30).

Despite these simplifications (concentration-independent |χ^(2)^|, |χ^(3)^|, cos(β); exponentially decaying potential), the extracted |χ^(3)^| spectra (Figure 7a) have a similar spectral shape with a maximum at ∼3200 cm^–1^ and a shoulder at ∼3400 cm^–1^. This spectral shape resembles the spectra for the bulk response of water near silica interfaces reported by others,^13,22,23^ although there is considerable spread in the reported spectral shapes for water near silica interfaces.^60^ Moreover, the |χ^(3)^| spectra agree well with the |χ^(3)^| spectra for water in contact with a charged lipid monolayer reported in ref (30) (dashed line in Figure 7a). Most importantly, the amplitude of the |χ^(3)^| spectra for all studied salts are nearly the same (e.g., the |χ^(3)^| values at the peak maxima in Figure 7a differ by <5% for the different salts), despite the largely different  values for the different salts (Figure 1). As such, the model in eq 7 predicts the χ^(3)^ response to be hardly affected by the nature of the ions. This insensitivity implies that the response of “bulk-like” water in the EDL is similar for all studied salts, which one would expect for the “bulk-like” response of aqueous salt solutions—in particular at low concentrations.

The interfacial |χ^(2)^| spectra (Figure 7b) vary with the nature of the ion. While we find a similar response in the presence of CsCl and KCl, the spectral magnitude at 3200–3400 cm^–1^ is somewhat enhanced for NaCl, and for LiCl, the response at these frequencies is further increased by ∼50%. The response for NaCl and LiCl is somewhat blue-shifted relative to the |χ^(3)^| spectra. We note that phase-resolved experiments have reported a red-shifted response of the interfacial water molecules.^22^ However, the experiments in ref (22) have been performed at high pH values where interfacial hydrogen-bonding may be enhanced due to the more negative surface charge, while our experiments were conducted at neutral pH. Although eq 7 does not fit the data very well for LiCl, we obtain the most pronounced changes in |χ^(2)^| for LiCl, which may point toward the enhanced response of water located at the very interface.

Within this model, we find the most pronounced changes with the nature of the cation for the relative phase between the interfacial and the bulk response [cos(β), Figure 7c]. For LiCl and NaCl, the phase difference between the two contributions is rather flat across the detected frequency range and can be as high as ∼120°, which is somewhat smaller than the 180 ± 20° suggested for NaCl.^14^ For KCl, the relative phase increases from ∼70° at low frequencies to ∼120° at high infrared frequencies. Such dispersion may, in fact, be expected, given that the χ^(3)^ and χ^(2)^ responses have dispersive line shapes themselves with a marked dispersion of the phases of the individual responses. For CsCl, we find the relative phases to be throughout <90°. As Φ(*z* = 0) < 0 for negatively charged silica, β < 90° indicates that for silica in contact with solutions of CsCl the χ^(3)^ and χ^(2)^ responses predominantly interfere destructively. As such, the model in eq 7 predicts the interference of the χ^(3)^ and χ^(2)^ responses to result in the markedly different magnitudes of the  curves (Figure 1) for the different salts: for LiCl, both terms interfere predominantly constructively, while for CsCl partial destructive interference reduces the detected *I*_SFG_ at all concentrations. These differences in phases will affect the phase of the overall SFG signal predominantly at high concentrations, where both contributions have a similar magnitude. At low concentrations, the phase is determined by the χ^(3)^ response and the phase of the response will only weakly depend on the nature of the ions, consistent with a previous phase-resolved SHG study that found the phase of the total signal is insensitive to the nature of the cation at 0.2 mmol/L,^11^ yet the phase varies with ionic strength.^10^

### Cation-Specific Response Is due to Near-Surface Water Orientation

**Figure 8:**
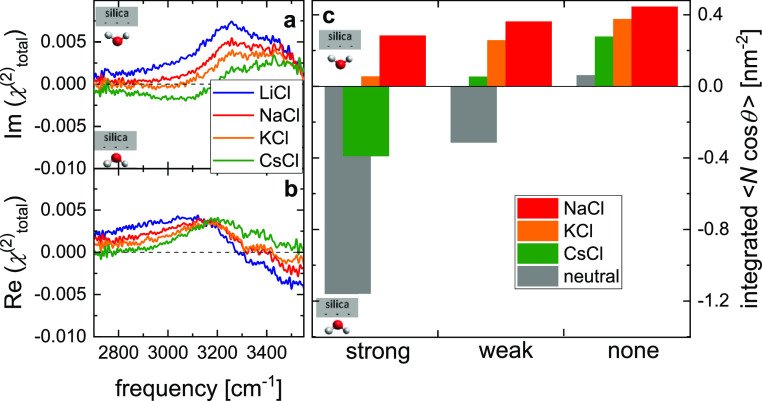
(a) Imaginary, , and (b) real,  SFG spectra for silica in contact with solutions of LiCl (blue), NaCl (red), KCl (orange), and CsCl (green) at 1 mol/L. (c) Integral over  from the center of the simulation box to the center of the silica of the orientation density profiles , where  is the number density of OH groups from both water and silica,  is the angle relative to the surface normal ( in the direction from the fluid to the surface), and the angular brackets denote averaging over the lateral dimensions. The OH groups are categorized as strongly, weakly, and non-hydrogen-bonded according to the criteria from ref (61). The data have been extracted from MD simulations of water in contact with silica with a surface charge density of 41 mC m^–2^ in the presence of 1 mol/L background salt solutions. Also shown are the results for an electroneutral silica interface in contact with pure water. See the Supporting Information for calculation details.

To evidence the ion-specificity of the phase of the spectral response in the near-surface region, we performed phase-resolved SFG experiments. Here, we focus on salt concentrations of 1 mol/L, for which the χ^(3)^ and χ^(2)^ contributions are predicted to have comparable magnitude: within the Gouy–Chapman approximation used for fitting eq 7 to the data, Φ(*z* = 0) ≈ −0.2 V. As such, the data in Figure 7 suggest |χ^(2)^| to be 20–50% of  and ion-specific differences in the phases of χ^(3)^ and χ^(2)^ contributions should be reflected in the phase of χ_total_^(2)^. Indeed, the phase-resolved experiments in Figure 8a,b demonstrate marked changes in the phase of χ_total_^(2)^ with variation in the nature of the cation (spectra in the absence of salt are shown in Figure S4, Supporting Information). While  is positive at 2700–3500 cm^–1^ in the presence of 1 mol/L LiCl, the spectra gradually vary with the increase in cation size, becoming negative for CsCl at 2800–3200 cm^–1^ (Figure 8a). This change in sign is consistent with the signatures of destructive interference as observed in the concentration-dependent SFG intensities (Figure 6) at low wavenumbers and suggests an ion-specific change in the orientation of the probed water molecules in the near-surface region—in line with what has been suggested for silica in contact with aqueous NaCl solutions.^23^

To elucidate the molecular-level origins of the ion-specificity of the phase of the spectra, we analyze the MD simulations of the 1 mol/L salt solutions in more detail: we categorize all OH groups according to the strength of the hydrogen-bond they donate (see the Supporting Infomation for details): strong (cf. red-shifted O–H stretch), weak, and non-bonded (cf. blue-shifted O–H stretch).^61^ To relate these different categories to the SFG experiment, for which an increased number density and/or enhanced average orientation of OH groups can enhance the signal strength, we determine their orientation density (cosine of the angle relative to the surface normal multiplied by their number density). The strongly H-bonded OH groups indeed point with the hydrogen atoms toward the bulk phase for CsCl (Figure 8c) and tend to be increasingly oriented toward the silica interface with the decrease in cation size (KCl, NaCl), consistent with the sign of the red-shifted  spectra (Figure 8a). The same trend is true for the weakly and non-bonded OH groups; however, these OH groups always point toward the interface, irrespective of the salt (Figure 8c). This orientation of the weakly and non-bonded OH groups is in line with the positive  at >3200 cm^–1^ observed for all salts. Note that due to the uncertainties related to the force-fields for Li^+^ (see Figure S3, Supporting Information), we do not analyze the orientation of water in the presence of LiCl. Yet, for CsCl, KCl, and NaCl, the MD simulations show that the near-surface orientation of water is the origin of the ion-specificity of the SFG features.

To understand this ion-specificity in the near-surface orientation of water, it is instructive to consider water in contact with the electroneutral interface: for uncharged silica, strongly bonded OH groups point toward the bulk (Figure 8c). For the charged interface in the presence of CsCl, the charge-induced change in orientation is insufficient to flip the orientation of these water molecules, whereas in the presence of KCl and NaCl, the effects of the surface charge suffice to alter the preferred orientation. This different orientation is intimately connected with the ionic displacement fields (Figure 5b): CsCl can efficiently screen the charge of the silica surface, and the intrinsic orientation of water in contact with neutral silica (with the hydrogen atoms pointing toward bulk water for strongly hydrogen-bonded water molecules) prevails. Changing the salt from CsCl via KCl to NaCl, the cations are increasingly located away from the interface, which is associated with a reduced screening of the interfacial charge. The resulting enhanced displacement field for KCl and NaCl compared to CsCl causes the water molecules to flip their orientation with their hydrogen atoms pointing toward the silica as they align according to this field. As such, the balance between the inherent water structure next to silica and ion-specific, charge-induced effects on water provides a rationale for our experimental findings.

Together, our results imply that it is challenging to use mean-field models to quantitatively model SFG experiments—in line with earlier findings^62^ —as the experiments do not solely reflect interfacial potentials. Our data demonstrate that the ion-specificity in Figure 1 can be largely explained by the near-surface response of water. The most dramatic changes with size of the cation can be explained by the orientation and corresponding phase of the optical response of near-surface water. This notion can provide a rationale for the variation of the detected SFG signals with the nature of the ions being highly sensitive to the pH of the solution,^20^ as  (de-)protonation of surface silanol groups can directly alter the hydrogen-bonded structure of water in this near-surface region. At neutral pH, this altered orientation results in a nearly complete cancellation of the ion-specific effects on the interfacial fields: we find the water structure to fully compensate for the ion-specific electric fields imposed by the ions, making the surface electrically invariant to the size of the cation in the aqueous solution.

## Conclusions

We have studied the ion-specificity of the response of water near a charged silica interface by interrogating the SFG response of water’s OH stretching band. The general shape of the measured SFG intensities as a function of ionic strength is only weakly sensitive to the size of the cation for solutions of CsCl, KCl, NaCl to LiCl. A comparison of the results for NaCl to experiments using NaI suggests that the shape of the curves is virtually unaffected upon changing the anion, and also, different polarization combinations of the experiment yield similar curves. Yet, in line with earlier studies, we find the SFG response to be markedly cation-specific: the magnitude of the response increases from CsCl to LiCl. Mean-field modeling based on the Poisson–Boltzmann equation indicates that sufficiently large variations in the surface potentials to explain the enhanced signals would require too drastic changes in the ion-adsorption or the permittivity of the solvent. Classical MD simulations support this notion as the simulations suggest the total surface electric field to be very similar for all salts. Taking both the surface field-induced response of water in the diffuse double layer and also the response of water bound to the silica interface into account, our data suggest that the size of the cation predominantly alters the phase of the signal generated by water molecules at the very interface. The different phase of the signals results in differently interfering SFG signals from the interfacial and the diffusive double layer. As this interference changes both magnitude and, to some extent, the slope of the  curves, it is challenging to directly relate the intensities in second-order spectroscopies to the surface potential. Phase-resolved SFG experiments, together with orientational analysis of the near-surface structure of water, show that the orientation of strongly hydrogen-bonded water is the cause of the ion-specific response: water adapts its orientation close to the surface to the (ion-specific) distribution of ions such that differences in the electric fields of the ions are nearly fully compensated for by the electric fields of water.

## Materials and Methods

### Sample Preparation

Lithium chloride (≥99.5%, Sigma-Aldrich), sodium chloride (≥99.5%, Roth), potassium chloride (≥99.5%, Sigma-Aldrich), cesium chloride (≥99.999%, Roth), and sodium iodide (99.6%, VWR) were used as received. The salts were dissolved in Milli-Q water and the concentration series was obtained from consecutive dilution. All sample solutions are measured at least 1 h after preparation to ensure CO_2_-equilibration. Electrolytes were measured in contact with a fused silica window (Korth Kristalle GmbH Infrasil 302, s/d: 60/40). The window was treated by UV–ozone cleaning for 30 min and stored in Milli-Q water until it is mounted on a flow cell (as described in ref (63)), which is then flushed with the sample solution for ∼5 min. The SFG spectra were recorded directly after switching off the flow. As the dissolution dynamics of silica in contact with aqueous solutions take place over tens of hours,^64^ the dissolution is too slow to influence the SFG results.

### SFG (Intensity) Spectroscopy

SFG spectra were recorded using an experimental setup based on a Ti:sapphire amplifier (Solstice Ace, Spectra Physics) that generates 800 nm pulses with a repetition rate of 1 kHz and femtosecond duration. Broadband infrared pulses (fwhm ∼ 400 cm^–1^) with 4 μJ are generated by a commercial optical parametric amplifier (TOPAS Prime, Spectra Physics) combined with a non-collinear difference frequency generation scheme. Visible pulses are generated by guiding the 800 nm pulses through a Fabry–Perot etalon (SLS Optics Ltd), resulting in spectrally narrowed (fwhm of ∼20 cm^–1^) pulses with ∼15 μJ pulse energy. The spectra are recorded via an electron multiplied charge-coupled device (emCCD) camera (ProEM 1600, Roper Scientific) attached to a spectrograph (Acton SpectraPro SP-2300, Princeton Instruments). All spectra were integrated for 10 min and recorded in either ssp (s-polarized SFG, s-polarized visible, and p-polarized IR) or pss (p-polarized SFG, s-polarized visible, and s-polarized IR) polarization combination with incident angles θ_vis_ ≈ 37° and θ_IR_ ≈ 33° of the visible and infrared pulse, respectively. At least two concentration series were recorded for each salt *I*_c_(ν,*c*). All data shown in the paper show the average of the individual series, each weighted by the maximum number of CCD counts. To compare the SFG intensities between the different salts, we performed reference experiments, where we subsequently measured 20 mmol/L solutions of CsCl, KCl, NaCl, and LiCl: *I*_ref_(ν). To take the frequency-dependent IR intensity of the infrared pulses into account, the *I*_ref_(ν) spectra are divided by the intensity spectra for a silica window with 100 nm chromium-free gold coating measured in ppp polarization combination. The SFG intensities of the spectra for each separately recorded *I*_c_(ν,*c*) series were then normalized by multiplying each experimental series with a frequency-dependent normalization factor *I*_ref_(ν)/*I*_c_(ν, 20 mmol/L).

### SFG (Phase-Resolved) Spectroscopy

Phase-resolved experiments were performed with a non-collinear beam geometry based on a Ti:sapphire amplified laser system (Spitfire Ace, Spectra-Physics). A part of the output was directed to a grating-cylindrical lens pulse shaper to produce a narrowband visible pulse (8 μJ pulse energy at the sample position, fwhm = ∼10 cm^–1^), while the other part was used to generate a broadband infrared (IR) pulse (3 μJ pulse energy, fwhm = ∼400 cm^–1^) through an optical parametric amplifier (Light Conversion TOPAS-C) combined with collinear DFG in a silver gallium disulfide (AgGaS_2_) crystal. The IR and visible beams were first focused onto a 200 nm thick ZnO on a 1 mm thick CaF_2_ window to generate a local oscillator (LO) signal in a similar manner to ref (65). Then, these beams were focused by two off-axis parabolic mirrors and overlapped spatially and temporally at the silica–aqueous solution interfaces. A fused silica glass plate with a 1.5 mm thickness was placed in the optical path for the LO signal in between the two off-axis parabolic, allowing the phase modulation for the LO signal. The visible, IR, and LO beams were refocused onto the sample interface with incident angles of 39, 33, and 37°, respectively. The SFG signal from the sample interfered with the SFG signal from the LO, generating the SFG interferogram, which was then dispersed in a spectrometer (Shamrock 303i, Andor Technology) and detected by an EMCCD camera (Newton, Andor Technology). To avoid sample drifts upon flowing electrolyte solutions, we used a height displacement sensor (CL-3000, Keyence). The sample’s tilt was also compensated upon changing solutions. For the silica samples, we used 2 mm thick silica windows partly coated with 100 nm thick gold film. During the measurement, the gold part was not in contact with the aqueous solution. Each spectrum was acquired with an exposure time of 30 min. The complex spectra of the second-order nonlinear susceptibility were obtained via Fourier analysis of the interferogram and normalization to that obtained from the silica–gold interface. We measured the complex spectra of the silica–gold interface by translating the sample stage. The variation of the thickness of the silica sample at different positions was found to be less than ∼0.5 μm, which has a negligible impact on the phase accuracy discussed here. We corrected the phase of all spectra such that the  spectrum of the silica–D_2_O interface at 3000–3400 cm^–1^ is zero.

### Molecular Dynamics Simulations

The silica is modeled using the force field developed by Emami et al.^48^ We use the Q3 form of silica, which has 4.7 silanol groups per nm^2^. We simulate at 5% deprotonation, corresponding to approximately pH 5, having a surface charge density of 0.26*e* nm^–2^ or 41 mC m^–2^. For water, we use the SPC/E model.^49^ The ion force fields are taken from Loche et al. for NaCl and KCl,^50^ from Fyta and Netz for CsCl (set 9),^52^ and from Horinek et al. for LiCl.^53^ A detailed comparison of the force-fields is shown in the Supporting Information. We truncate the Lennard-Jones interaction at 0.9 nm. The Coulomb interactions are truncated in real space at 0.9 nm, with long-range interaction being handled using particle mesh Ewald summation.^66^ The length of bonds involving hydrogen atoms are constrained using the LINCS algorithm. The temperature is fixed at 300 K using the v-rescale algorithm, and the pressure at 1 bar in the direction perpendicular to the surface using the anisotropic Berendsen barostat using the compressibility of water of 4.5 × 10^–5^ bar^–1^. The system contains 2500 water molecules and either 6 cations (0 mol/L), 11 cations and 5 anions (0.1 mol/L), or 51 cations and 45 anions (1 mol/L). The lateral size is 3.3 × 3.5 nm and the size in *z*-direction is between 9.3 and 9.5 nm, depending on the salt type and concentration. Every system is simulated for 250 ns using a 2 ps time step. Electric fields are directly calculated from the average charge distribution (see eq 6). The electrochemical potential is calculated from the double integral over the total charge density. The profile is averaged over the two symmetric halves of the water slab. The integration constant (potential in the center of the slab) is determined from the solution to the Gouy–Chapman equation. The orientation density is calculated as the volume density of OH groups weighted by cos θ. Every OH group is categorized according to its hydrogen bond status according to criteria based on the potential of mean force (see also Supporting Information).^61^ The profiles are averaged over the two symmetric halves and integrated over *z* to produce Figure 8c.

## Supporting Information Available

Integrated SFG intensities for different polarization combinations and anions, detailed comparison of different ion force-fields, details on the calculation of the orientational densities, and phase-resolved SFG spectra in the absence of electrolyte (PDF)
