# Supplementary material for: Nature of Cations Critically Affects Water at the Negatively Charged Silica Interface
Source: J Am Chem Soc. 2022 Oct 23;144(43):19726–38. doi: 10.1021/jacs.2c02777 (PMC9634801; doi:10.1021/jacs.2c02777)
Supplement: Supplementary file 1 — ja2c02777_si_001.pdf [file ja2c02777_si_001.pdf]

SUPPORTING INFORMATION

for

Nature of cations critically affects water at the negatively charged silica interface

Johannes Hunger,[1*] Jan Schäfer,[1] Patrick Ober,[1] Takakazu Seki,[1] Yongkang Wang,[1] Leon Prädel,[1] Yuki Nagata,[1] Mischa Bonn,[1] Douwe Jan Bonthuis,[2,*] Ellen H. G. Backus[1,3,*]

[1]Department for Molecular Spectroscopy, Max Planck Institute for Polymer Research, Ackermannweg 10, 55128 Mainz, Germany

[2] Institute of Theoretical and Computational Physics, Graz University of Technology, Petersgasse16/II, 8010 Graz, Austria

[3] University of Vienna, Faculty of Chemistry, Institute of Physical Chemistry, Währinger Strasse 42, 1090 Vienna, Austria

Anion and polarization effect on integrated SFG intensities

To further explore the effect of polarization combinations, *ssp* or *pss*, and anion, NaCl or NaI, we also compare the SFG intensities, integrated from 2920 to 3470 cm^{-1}. For better comparison, the integrated intensities were normalized to the integrated SFG intensity in the absence of salt. These data in Figure S1 show that, similar to the data at 3150 cm^{-1} shown in Figure 2 of the main manuscript, also the variation of the integrated intensity with concentration is rather insensitive to the polarization combination and to the anion.

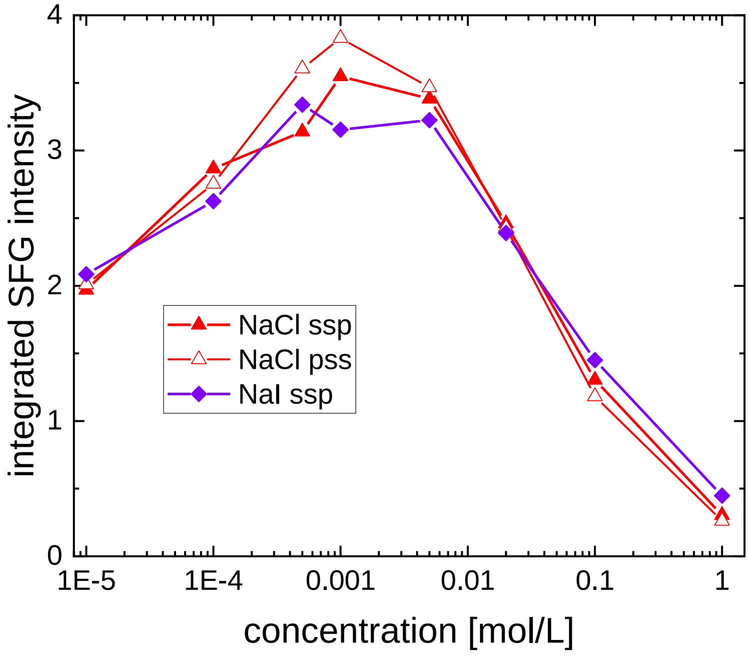

Figure S1: Integrated O-H-stretch SFG intensity at the silica/water interface with varying salt concentration. Data for NaCl with *ssp* polarization combination (solid red symbols), compared to NaCl with *pss* polarized beams (open red symbols), and to NaI with ssp polarization combinations (purple symbols). Intensity spectra were normalized to the intensity spectra of silica in contact with gold. Integrated spectra were normalized to the integrated intensity in the absence of salt.

Comparison of ion force fields

We have tested several different ionic force-fields, the parameters of which are listed in Table S1. The ion force-fields from Loche *et al.* are based on a simultaneous optimization of NaCl, NaBr, KCl and KBr for solvation free energy and activity.[1] The Lennard-Jones interactions and real-space Coulomb interactions are truncated after 0.9 nm, with long range Coulomb interaction being handled using particle mesh Ewald summation.[2] All other ion force-fields are based on the NaCl force field from Smith and Dang,[3,4] after which Cs^+ was optimized to reproduce the solvation free energy and the activity coefficients in bulk water.[5] Li^+ was optimized to reproduce the solvation free energy and enthalpy.[6]

As can be seen from Figure S2, and as concluded in the main manuscript, the total interfacial electric fields are insensitive to the nature of the ions and also to the exact choice of the force-fields. However, the ions' distributions markedly depend on the force-field parameters as evident from the ionic displacement fields shown in Figure S2. These different ion distributions also affect the orientation of water (see water electric field in Figure S2), relevant to the analysis of the SFG results in the main manuscript.

For the choice of the force-field parameters for the simulations shown in the main manuscript, we use reproduction of the experimental solvation free energy, as well as the agreement of the simulated activity coefficients and the experimentally determined activity coefficients as criteria. For NaCl and KCl, it has been shown[1] that the force-fields reported by Loche et al[1] together with SPC/E water can excellently reproduce these experimental quantities. As we show in Figure S3, this is also true for CsCl using the force-field reported by Fyta (set 9, Table S1).[5] Therefore, all data shown in the main manuscript are based on these force-fields. For LiCl, we use the force-fields reported by Horinek, which were optimized to reproduce the solvation free energy and entropy.[6] As we show in Figure S3, this optimization results in an underestimation of the thermodynamic activity coefficients for both force-fields. Set 5a for LiCl is closer to the experimental values. Hence, we show in the main manuscript data obtained using this force-field. Yet, also set 5a underestimates thermodynamic activity (Figure S3b). This underestimation indicates that ion-ion interactions are overestimated, which may also result in an overestimation of the adsorption to the silica interface. As such, the ion distributions for LiCl shown in the main manuscript should be interpreted with caution, and we refrain from including these simulations into our orientational analysis (Figure 8 of the main manuscript).

Table S1: Force field parameters. Lorentz-Berthelot combination rules are used for all cross combinations σ_{ij} and ε_{ij}.

ion	force field	σ_{ii} [nm]	ε_{ij} [kJ/mol]	ref
Li^+	Dang	0.1506	0.6904	7
Li^+	Horinek set 5	0.2870	$6.1 \cdot 10^{-4}$	6
Li^+	Horinek set 5a	0.1370	1.5400	6
Na^+	Dang	0.2583	0.4186	3
Na^+	Loche	0.2310	0.4500	1
K^+	Dang	0.3154	0.4186	8
K^+	Fyta	0.2690	2.4400	5
K^+	Loche	0.2830	0.9000	1
Cs^+	Fyta set 6	0.3491	0.3250	5
Cs^+	Fyta set 9	0.3331	1.5400	5
Cs^+	Horinek set 5	0.5170	$6.1 \cdot 10^{-4}$	6
Cs^+	Dang	0.3884	0.4186	3
Cl^-	Horinek, Dang, Fyta	0.4400	0.4186	3,5,6
Cl^-	Loche	0.4300	0.4200	1

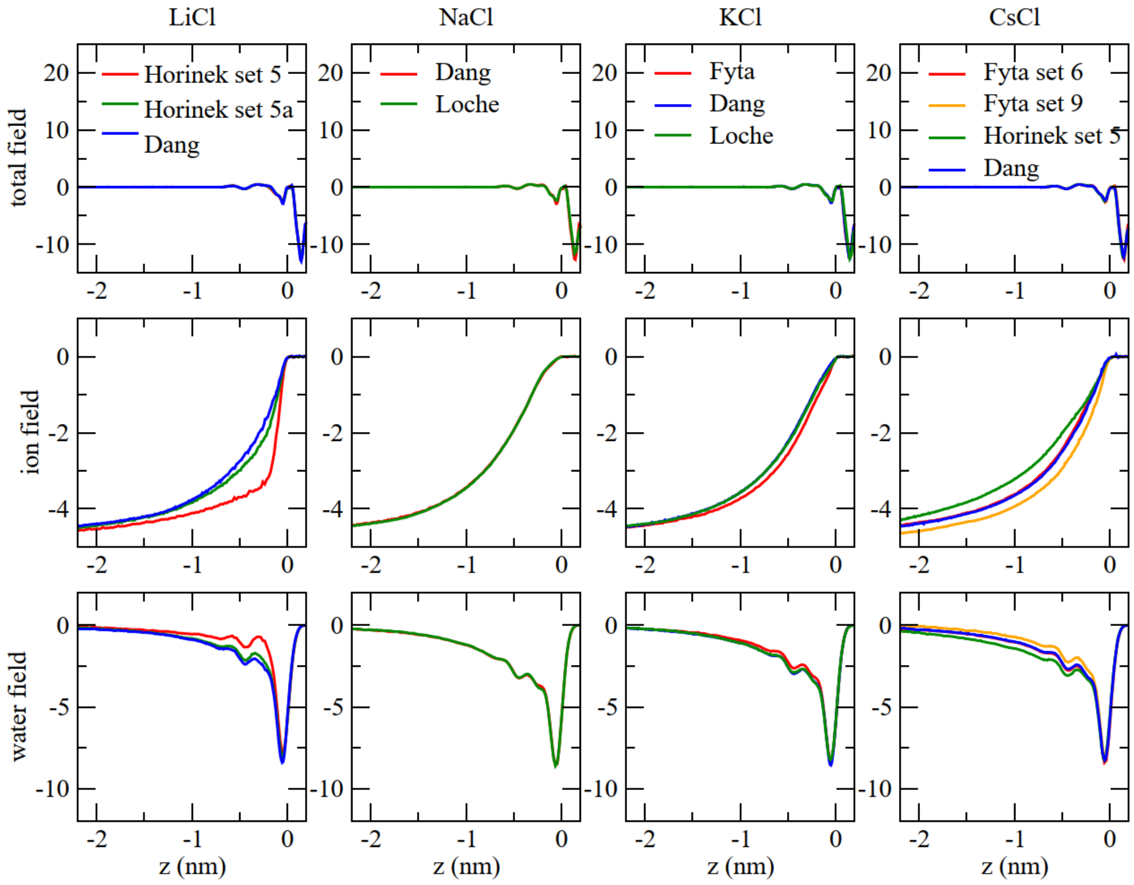

Figure S2: Total electric field (top row) and contributions from ions (center row), and water (bottom row) at a bulk concentration of 0.1 mol/L using different force-fields for LiCl, NaCl, KCl, and CsCl from left to right.

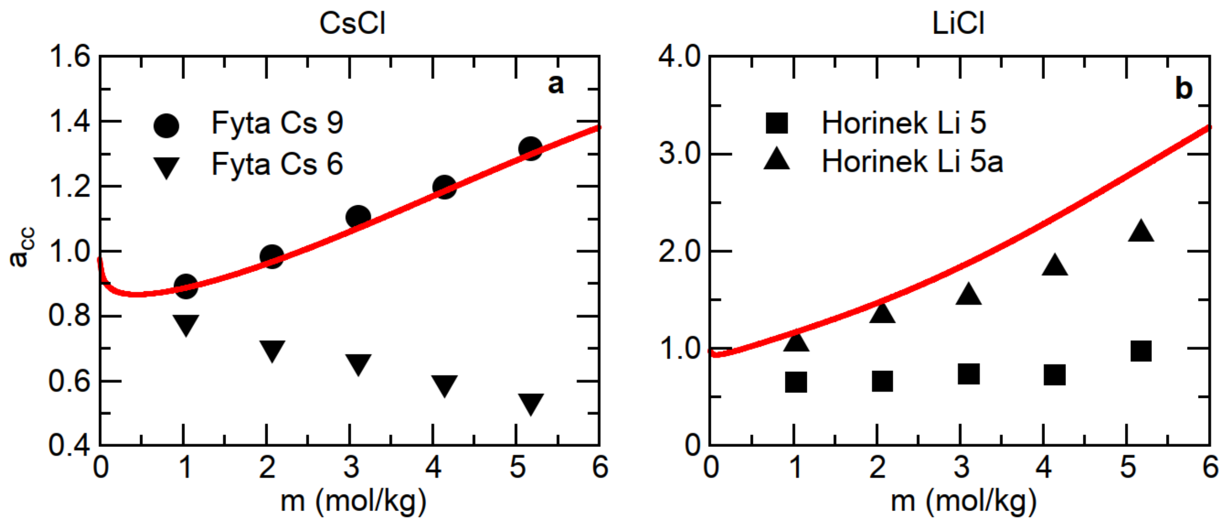

Figure S3: (a) Activity derivative[1] for CsCl using the force-fields reported by Fyta (set 6 and set 9)[5] as a function of concentration. (b) Activity derivative[1] for LiCl using the force-fields reported by Horinek (set 5 and set 5a)[6] as a function of concentration. Symbols show simulated activity coefficients and solid lines show experimental values.[9,10]

Calculation of the orientation density

For every OH group of both water and silanol, we determine the third atom X nearest to the H and calculate the OHX angle α_{OHX} and the HX distance δ_{HX}. To determine the hydrogen bond conditions, we use the criteria from Ref. 11 for TIP4P/2005 water. That means that we consider a hydrogen bond to be formed if

$$\frac{(\delta_{\mathrm{HX}}-\delta_{\mathrm{c}})^2}{\delta_{\mathrm{r}}^2}+\frac{(\alpha_{\mathrm{OHX}}-\alpha_{\mathrm{c}})^2}{\alpha_{\mathrm{r}}^2}\leq 1 \text{ and } \delta_{\mathrm{HX}}<\delta_{\mathrm{t}}.$$

The parameters of the strong hydrogen bond (potential of mean force below $-2k_{\mathrm{B}}T$)[11] are

$\delta_{\mathrm{c}} = 0.19506$ nm

$\delta_{\mathrm{r}} = 0.036933$ nm

$\delta_{\mathrm{t}} = 0.208827$

$\alpha_{\mathrm{c}} = -2.42805\,^\circ$

$\alpha_{\mathrm{r}} = 31.87969\,^\circ$

and for the weak hydrogen bond (potential of mean force between $0k_{\mathrm{B}}T$ and $-2k_{\mathrm{B}}T$)[11]

$\delta_{\mathrm{c}} = 0.226221$ nm

$\delta_{\mathrm{r}} = 0.072934$ nm

$\delta_{\mathrm{t}} = 0.234899$

$\alpha_{\mathrm{c}} = -1.13282\,°$

$\alpha_{\mathrm{r}} = 46.85052\,°$.

For all atoms X which are not a water oxygen itself, the values of δ_{c} and δ_{t} are changed to δ_{c}' and δ_{t}' based on the geometry of the bond,

$$\delta_{\mathrm{c}}' = \delta_{\mathrm{c}} - \sqrt{\sigma_{\mathrm{OO}}^2 - \delta_{\mathrm{OH}}^2 sin^2\alpha_{\mathrm{OHX}}} + \sqrt{\sigma_{\mathrm{OX}}^2 - \delta_{\mathrm{OH}}^2 sin^2\alpha_{\mathrm{OHX}}}$$

$$\delta_{\mathrm{t}}' = \delta_{\mathrm{t}} - \sqrt{\sigma_{\mathrm{OO}}^2 - \delta_{\mathrm{OH}}^2 sin^2\alpha_{\mathrm{OHX}}} + \sqrt{\sigma_{\mathrm{OX}}^2 - \delta_{\mathrm{OH}}^2 sin^2\alpha_{\mathrm{OHX}}}$$

where the Lennard-Jones parameter of the interaction between water oxygen and itself is given by σ_{OO} and of the interaction between the oxygen of the OH group and X is given by σ_{OX} (where O can be either the silanol or water oxygen). Any OH group that does not fulfil the hydrogen bond criterion with either of the parameter sets is considered non-bonded.

For every category of OH groups, we calculate the volumetric number density profile as a function of the distance to the surface weighted by the cosine of the angle between the OH bond vector and the vector normal to the silica surface. That means that OH vectors pointing straight toward the surface contribute 1, OH vectors oriented parallel do not contribute, and OH vectors pointing straight toward the bulk contribute -1 to the orientation density. Figure 8c in the main text shows the orientation density integrated over the coordinate z from the center of the water slab to the center of the silica.

Phase-resolved SFG spectrum of the neat silica-water interface

For reference, we have also determined the phase-resolved SFG spectrum of the bare silica-water interface (in the absence of added salts). In Figure S4 we compare this spectrum to the spectra in the presence of 1 mol/L salts (also shown in Figure 8a&b of the main manuscript). The spectrum of the bare silica-water interface agrees well with the spectrum reported by Tahara et al.[12]

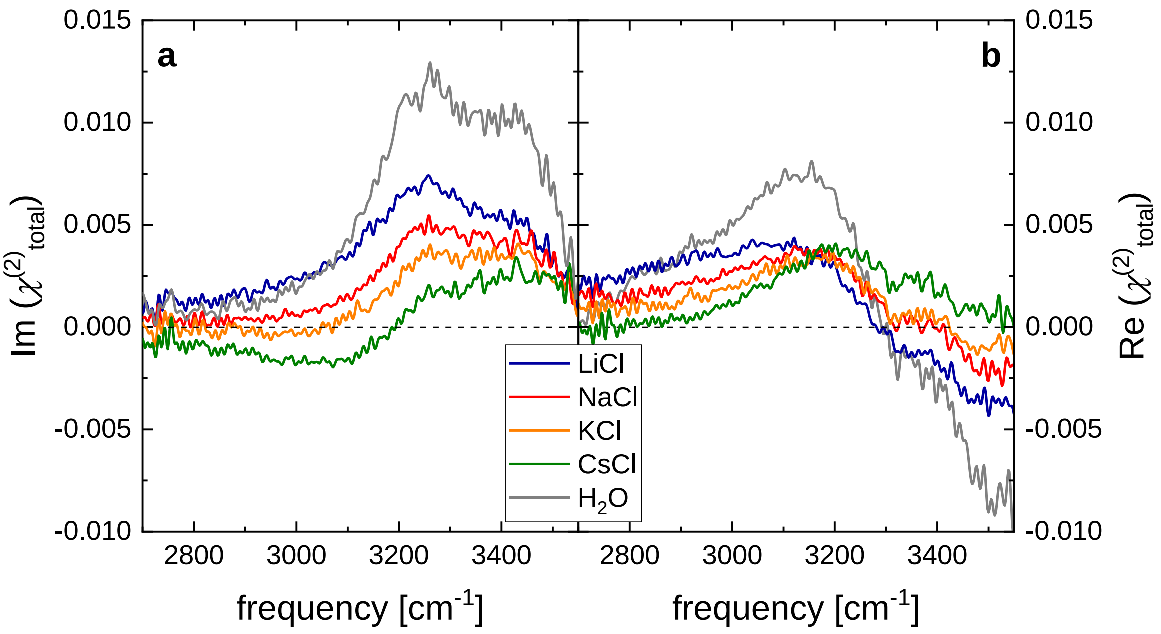

Figure S4: (a) Imaginary, $Im(\chi^{(2)}{}_{total})$, and (b) real, $Re(\chi^{(2)}{}_{total})$, SFG spectra for silica in contact with solutions of LiCl (blue), NaCl (red), KCl (orange), CsCl (green) at 1 mol/L, and to neat water (in the absence of added salt, grey).

Supporting References

(1) Loche, P.; Steinbrunner, P.; Friedowitz, S.; Netz, R. R.; Bonthuis, D. J. Transferable Ion Force Fields in Water from a Simultaneous Optimization of Ion Solvation and Ion–Ion Interaction. *J. Phys. Chem. B* **2021**, *125* (30), 8581–8587. https://doi.org/10.1021/acs.jpcb.1c05303.

(2) Allen, M. P.; Tildesley, D. J. *Computer Simulation of Liquids*; Clarendon Press, 1989.

(3) Dang, L. X. Mechanism and Thermodynamics of Ion Selectivity in Aqueous Solutions of 18-Crown-6 Ether: A Molecular Dynamics Study. *J. Am. Chem. Soc.* **1995**, *117* (26), 6954–6960. https://doi.org/10.1021/ja00131a018.

(4) Smith, D. E.; Dang, L. X. Computer Simulations of NaCl Association in Polarizable Water. *J. Chem. Phys.* **1994**, *100* (5), 3757–3766. https://doi.org/10.1063/1.466363.

(5) Fyta, M.; Netz, R. R. Ionic Force Field Optimization Based on Single-Ion and Ion-Pair Solvation Properties: Going beyond Standard Mixing Rules. *J. Chem. Phys.* **2012**, *136* (12), 124103. https://doi.org/10.1063/1.3693330.

(6) Horinek, D.; Mamatkulov, S. I.; Netz, R. R. Rational Design of Ion Force Fields Based on Thermodynamic Solvation Properties. *J. Chem. Phys.* **2009**, *130* (12), 124507. https://doi.org/10.1063/1.3081142.

(7) Dang, L. X. Development of Nonadditive Intermolecular Potentials Using Molecular Dynamics: Solvation of Li^+ and F^- Ions in Polarizable Water. *J. Chem. Phys.* **1992**, *96* (9), 6970–6977. https://doi.org/10.1063/1.462555.

(8) Chang, T.-M.; Dang, L. X. Detailed Study of Potassium Solvation Using Molecular Dynamics Techniques. *J. Phys. Chem. B* **1999**, *103* (22), 4714–4720. https://doi.org/10.1021/jp982079o.

(9) Hamer, W. J.; Wu, Y. Osmotic Coefficients and Mean Activity Coefficients of Uni- univalent Electrolytes in Water at 25°C. *J. Phys. Chem. Ref. Data* **1972**, *1* (4), 1047–1100. https://doi.org/10.1063/1.3253108.

(10) Lide, D. R. *CRC Handbook of Chemistry and Physics*, 85th ed.; CRC Press: Boca Raton, USA, 2005.

(11) Muthachikavil, A. V.; Peng, B.; Kontogeorgis, G. M.; Liang, X. Distinguishing Weak and Strong Hydrogen Bonds in Liquid Water—A Potential of Mean Force-Based Approach. *J.*

Phys. Chem. B **2021**, *125* (26), 7187–7198. https://doi.org/10.1021/acs.jpcb.1c02816.

(12) Myalitsin, A.; Urashima, S. H.; Nihonyanagi, S.; Yamaguchi, S.; Tahara, T. Water Structure at the Buried Silica/Aqueous Interface Studied by Heterodyne-Detected Vibrational Sum-Frequency Generation. *J. Phys. Chem. C* **2016**, *120* (17), 9357–9363. https://doi.org/10.1021/acs.jpcc.6b03275.
